# Selective and Irreversible Inhibitors of Mosquito Acetylcholinesterases for Controlling Malaria and Other Mosquito-Borne Diseases

**DOI:** 10.1371/journal.pone.0006851

**Published:** 2009-08-28

**Authors:** Yuan-Ping Pang, Fredrik Ekström, Gregory A. Polsinelli, Yang Gao, Sandeep Rana, Duy H. Hua, Björn Andersson, Per Ola Andersson, Lei Peng, Sanjay K. Singh, Rajesh K. Mishra, Kun Yan Zhu, Ann M. Fallon, David W. Ragsdale, Stephen Brimijoin

**Affiliations:** 1 Molecular Pharmacology and Experimental Therapeutics, Mayo Clinic, Rochester, Minnesota, United States of America; 2 Swedish Defence Research Agency, CBRN Defence and Security, Umeå, Sweden; 3 Department of Chemistry, Kansas State University, Manhattan, Kansas, United States of America; 4 Department of Entomology, Kansas State University, Manhattan, Kansas, United States of America; 5 Department of Entomology, University of Minnesota, Saint Paul, Minnesota, United States of America; East Carolina University, United States of America

## Abstract

New insecticides are urgently needed because resistance to current insecticides allows resurgence of disease-transmitting mosquitoes while concerns for human toxicity from current compounds are growing. We previously reported the finding of a free cysteine (Cys) residue at the entrance of the active site of acetylcholinesterase (AChE) in some insects but not in mammals, birds, and fish. These insects have two AChE genes (AP and AO), and only AP-AChE carries the Cys residue. Most of these insects are disease vectors such as the African malaria mosquito (*Anopheles gambiae* sensu stricto) or crop pests such as aphids. Recently we reported a Cys-targeting small molecule that irreversibly inhibited all AChE activity extracted from aphids while an identical exposure caused no effect on the human AChE. Full inhibition of AChE in aphids indicates that AP-AChE contributes most of the enzymatic activity and suggests that the Cys residue might serve as a target for developing better aphicides. It is therefore worth investigating whether the Cys-targeting strategy is applicable to mosquitocides. Herein, we report that, under conditions that spare the human AChE, a methanethiosulfonate-containing molecule at 6 µM irreversibly inhibited 95% of the AChE activity extracted from *An. gambiae* s. str. and >80% of the activity from the yellow fever mosquito (*Aedes aegypti* L.) or the northern house mosquito (*Culex pipiens* L.) that is a vector of St. Louis encephalitis. This type of inhibition is fast (∼30 min) and due to conjugation of the inhibitor to the active-site Cys of mosquito AP-AChE, according to our observed reactivation of the methanethiosulfonate-inhibited AChE by 2-mercaptoethanol. We also note that our sulfhydryl agents partially and irreversibly inhibited the human AChE after prolonged exposure (>4 hr). This slow inhibition is due to partial enzyme denaturation by the inhibitor and/or micelles of the inhibitor, according to our studies using atomic force microscopy, circular dichroism spectroscopy, X-ray crystallography, time-resolved fluorescence spectroscopy, and liquid chromatography triple quadrupole mass spectrometry. These results support our view that the mosquito-specific Cys is a viable target for developing new mosquitocides to control disease vectors and to alleviate resistance problems with reduced toxicity toward non-target species.

## Introduction

Mosquitoes are a principal insect vector of infectious diseases that afflict both developing countries and industrialized nations. For example, *Anopheles gambiae* sensu stricto transmits malaria in Sub-Saharan Africa [1], *Culex pipiens* L. transmits St. Louis encephalitis [2] and West Nile virus [3], and *Aedes aegypti* L. transmits dengue, yellow fever, and chikungunya [4]. Recently, mosquito populations have surged due to emergence of insect populations with increased resistance to common insecticides. Another important factor in pest resurgence is lax control measures owing partly to growing concerns about insecticide safety [5]. Such concern may be well founded, since many insecticides phosphorylate or carbamylate indiscriminately a ubiquitous catalytic serine residue at the active site of acetylcholinesterase (AChE, EC 3.1.1.7), a serine hydrolase vital for regulating cholinergic neurotransmission in mammals, birds, fish, and insects [6]. Thus, there is an urgent need for novel insecticides to control mosquito-borne diseases, especially malaria. According to World Malaria Report 2008 (http://apps.who.int/malaria/wmr2008/), half of the world's population is at risk of malaria, and an estimated 247 million cases led to nearly 881,000 deaths in 2006.

Unlike mammals, many disease-transmitting or crop-pest insects have two AChE genes (AP and AO) [7]–[12]. Interestingly, a free cysteine (Cys) residue, for example, Cys286 of *An. gambiae* s. str. AP-AChE (*Ag*AP-AChE), is present at the entrance to the active site of insect AP-AChEs but not at that of AO-AChEs and AChEs from mammals, birds, and fish [13]–[15]. In addition, mosquitoes have an additional arginine residue (Arg339 of *Ag*AP-AChE) at the rim of the AP-AChE active site that appears to be genus-specific [14]. Recently we reported that a methanethiosulfonate-containing molecule designed to target the active-site Cys residue irreversibly inhibited all AChE activity extracted from aphids while an identical exposure caused no effect on the human AChE [16]. We also reported that the irreversible inhibition is primarily caused by the formation of a disulfide bond between the inhibitor and the Cys residue as evident from the reversal of inhibition by 2-mercaptoethanol, a disulfide-bond-breaking agent [16]. These results show that AP-AChE is responsible for most of the acetylcholine-hydrolyzing activity in aphids, and they prove the concept that the active-site Cys residue in aphid AP-AChE is a viable target for developing insect-selective pesticides to control crop damage caused by aphids.

In this context, it appears worth exploring new inhibitors that target two mosquito-specific residues (Cys286 and Arg339 of *Ag*AP-AChE or their equivalents of other mosquito species) to control mosquito-borne diseases. Such agents can alleviate the resistance problem because they are novel and because the targeted residues stabilize the active-site conformation [14] and hence are relatively resistant to selective pressure. Equally important, these chemicals should be less toxic to mammals than current anticholinesterases that attack the ubiquitous catalytic serine residue of all AChEs.

On the other hand, it has been reported that iodoacetamide-containing AChE inhibitors do not bond covalently to *Ag*AP-AChE [17]. Since iodoacetamide is a commonly used sulfhydryl agent, such a finding would suggest that the active-site Cys residue in *Ag*AP-AChE is not targetable, a conclusion advanced in a recent reconsideration of this issue [18]. Further questions regarding Cys-targeting inhibitors arise from our own observation that such inhibitors can inhibit the human AChE (*h*AChE) in irreversible fashion, although this enzyme has no Cys residue near its active site [16]. Therefore, a re-investigation of the extent and mechanisms of irreversible inhibition of *Ag*AP- and mammalian AChEs by sulfhydryl agents is required before pursuing mosquitocides that target the two mosquito-specific residues.

In this article, we report data confirming that Cys286 of *Ag*AP-AChE or its equivalent in the *Ae. aegypti* or *C. pipiens* is targetable by sulfhydryl agents and that AP-AChE contributes most of the measurable AChE activity in *An. gambiae* s. str., *Ae. aegypti*, and *C. pipiens*. Our studies also offer new insights into irreversible inhibition mechanisms for AChEs from different species. The results support our notion that the mosquito-specific Cys is a viable target for new mosquitocides to control disease vectors and to alleviate resistance problems with reduced toxicity toward non-target species.

## Results

### 2.1. Irreversible Inhibition of Mosquito AChEs

**Figure 1:**
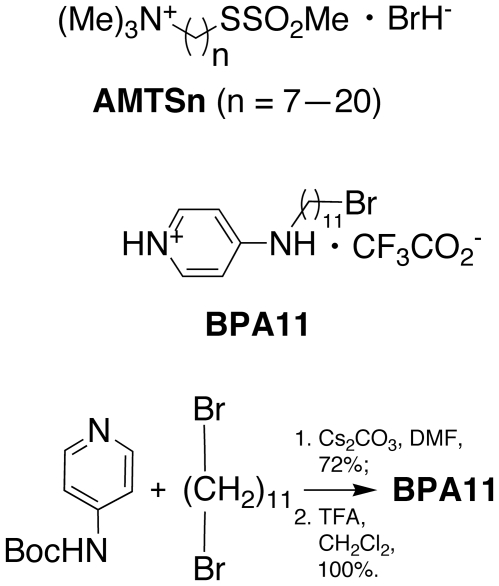
Chemical structures of AMTS7–AMTS20 and BPA11 and a synthetic scheme for BPA11.

To investigate whether the active-site Cys residue of mosquito AP-AChE can be targeted by sulfhydryl agents and to determine how much AP-AChE contributes to AChE activity in mosquito, we tested the inhibition of total AChE activity in extracts from three mosquito species using our recently reported assay protocol and active-site-Cys-targeting inhibitors, **AMTS7**–**AMTS20** [16] and **BPA11** (see Figure 1). At 6 µM, **AMTS17**–**AMTS20** are known to irreversibly inhibit aphid AChEs but not *h*AChE [16]. **AMTSn** have a trimethylammonium group on one end of an alkylene chain to confer affinity through a cation-pi interaction with Trp87 at the active site [19], [20] and a methanethiosulfonate group on the other end to form an adduct preferentially with a free cysteine residue [21]. Based on the same design principle, **BPA11** was made according to the scheme in Figure 1 to replace the trimethylammonium and methanethiosulfonate groups of **AMTSn** with 4-amino-pyridinium and bromide, respectively, for the purpose of retaining affinity while reducing reactivity toward Cys residues “in general” (*i.e.*, to minimize interactions with non-target SH-containing proteins).

*An. gambiae* s. str., *Ae. aegypti*, and *C. pipiens* were selected for this study on the basis of disease relevance, local prevalence, and their AP-AChE sequences that contain the mosquito-specific residues of Cys and Arg at the rim of the active site [14], [16]. Crude extracts rather than purified or recombinant AP-AChEs were used to delineate the relative role of AP-AChE in insect AChE activity. AChE inhibition assays on limited samples were enabled by a method that measures hydrolysis of ^3^H-acetylcholine [22] with a sensitivity that allowed 200 determinations with a 10∶1 signal-to-blank ratio from a single mosquito. Importantly, this radiometric assay precluded false inhibition and other interference that could arise with the standard Ellman method [23] owing to a reaction between the substrate, thiocholine, and the Cys-targeting sulfhydryl reagents under study.

**Figure 2:**
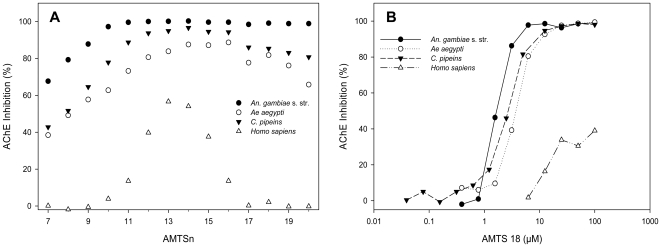
Effects of inhibitor length and concentration on AChE inhibition by AMTSn. (A) Irreversible AChE inhibition determined in extracts from *An. gambiae* s. str., *Ae. Aegypti, C. pipiens*, and human RBCs exposed to 6 µM AMTSn followed by overnight dialysis. (B) Dose response analysis of irreversible inhibition by AMTS18 in three mosquito species and *h*AChE.

We exposed a mosquito extract to each **AMTSn** at 6 µM for 1 hour and then dialyzed overnight against a large excess of 0.09% NaCl and 0.1 M sodium phosphate at pH of 7.4 to remove the free inhibitor before assay of residual AChE activity using the radiometric assay (see Section 4.3). Analysis of inhibition as a function of compound length revealed a broad peak of AChE inhibitory activity with a maximal inhibition near 100% in the case of *An. gambiae* s. str. and >80% in the case of *Ae. aegypti* or *C. pipiens* (Figure 2A). The AChEs from all three species were maximally inhibited by mid-length inhibitors (**AMTS12**–**AMTS15**), but for each species the long-chain inhibitors (**AMTS16**–**AMTS20**) had the greatest selectivity of mosquito AChE over *h*AChE. Consistently, 1 µM **BPA11**, an inherently less reactive molecule of similar effective length, also caused selective and irreversible inhibition of *Ag*AChE after a 2.5-hour incubation, although much lesser in extent (10%).

### 2.2. Slow, Partial, and Irreversible Inhibition of the Human AChE

Although **AMTS17**–**AMTS20** at 6 µM had shown no irreversible inhibition of *h*AChE in our previously reported studies [16], a full dose-response analysis detected irreversible inhibition of *h*AChE at inhibitor concentrations of >10 µM (Figure 2B). The inhibitory effect on *h*AChE by **AMTS17**–**AMTS20** at 100 µM is similar to that previously reported at 6 µM for the less selective compound, **AMTS13** [16]. Likewise, when the **BPA11** concentration was increased from 1 to 100 µM, the irreversible inhibition of *h*AChE rose from 0 to 30%. In the **AMTSn** series at a concentration of 100 µM, the irreversible inhibition of *h*AChE decreased with increasing chain length. Corresponding dose-response studies of the longer-chain molecules with each mosquito species showed that the concentration required to irreversibly inhibit mosquito AChE by 50% was ∼100-fold lower than required to inhibit *h*AChE equivalently. The optimal combination of potency and selectivity was achieved with **AMTS17**.

### 2.3. Characterization of Irreversible Inhibitions of *Ag*AChE and *h*AChE

The irreversible inhibition of *h*AChE by **AMTSn** at high concentrations prompted further studies to confirm that the irreversible inhibition of mosquito AChE by **AMTSn** is due to the conjugation of the inhibitor to the active-site Cys residue and that the irreversible mosquito AChE inhibition is mechanistically different from the irreversible inhibition of *h*AChE. We characterized the irreversible inhibition of *Ag*AChE and *h*AChE by **AMTS13** or **AMTS18** at 6 µM, as inhibitor concentrations of >6 µM are less relevant to insecticide development. These inhibitors were chosen because, as reported previously [16], **AMTS18** and **AMTS13** showed 0% and 57% irreversible inhibition of *h*AChE, respectively, at 6 µM with one-hour exposure followed by prolonged dialysis.

In search of evidence for conjugation to the active-site Cys residue, we found that 2-mercaptoethanol caused significant reactivation of the **AMTS13**-inhibited *Ag*AChE (data not shown) and the **AMTS18**-inhibited *Ag*AChE (Table 1), although it concomitantly reduced the catalytic activity of the *apo Ag*AChE. The latter was presumably due to disulfide bond reduction in AChE by 2-mercaptoethanol (see Section 3.1.1). In contrast, the *h*AChE activity inhibited by **AMTS18** was not restored by 2-mercaptoethanol (Table 1), nor by pralidoxime [24], a commonly used agent for regenerating a free catalytic serine residue of AChE (data not shown). These results indicate that the irreversible inhibition of mosquito AChE is mechanistically different from that of *h*AChE.

**Table 1:** Partial Reactivation of the AMTS18-inhibited *Ag*AChE by 2-Mercaptoethanol (2ME), Which Reverses the AMTS18 Inhibition and Concomitantly Denatures *Ag*AChE.

Pretreatment	Treatment	Incubation Time (hr)	Raw *Ag*AChE activity (cpm)	% *Ag*AChE activity reduced by 2ME and/or AMTS18	% *Ag*AChE activity recovered by 2ME
None	None	2	8400	0	
”	None	6	5130	0	
”	2ME	2	5900	30	
”	2ME	6	4500	12	
**AMTS18**	None	2	25	100	0
”	None	6	11	100	0
”	2ME	2	894	89	15
”	2ME	6	1400	73	31

The *An. gambiae* s. str. extracts were exposed to **AMTS18** (6 µM) for 1 hour and/or to 2ME (100 mM) for different periods of time. After the exposure(s), samples were dialyzed overnight and the AChE activity was measured. Activities are mean values of triplicate determinations expressed as percentages of the *Ag*AChE activity.

**Figure 3:**
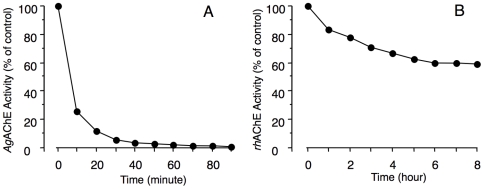
Time courses of irreversible inhibition for mosquito and human AChEs by AMTS13. Extracts of *An. gambiae* s. str. larva and *rh*AChE were exposed, respectively, to 6 µM AMTS13 at room temperature for defined intervals of time terminated by addition of glutathione (to a final concentration of 500 µM). Shown is AChE activity in treated samples assayed radiometrically.

In additional studies we observed that glutathione conjugated to **AMTS13** within seconds while by itself having no detectable effect on AChE activity in extracts from mosquitoes, human red blood cells (RBCs), or recombinant *h*AChE (*rh*AChE). The rapid conjugation enabled us to determine the time course of the irreversible AChE inhibition by adding excess glutathione to a reaction solution to remove the free inhibitor abruptly and completely (control experiments revealed no sign of reactivation when fully inhibited enzyme was exposed to glutathione). Such time-course studies showed that 6 µM **AMTS13** caused rapid irreversible inhibition of *Ag*AChE, with a half-time less than 10 minutes and nearing completion within 30 minutes (Figure 3A). By contrast, a four-hour incubation with the same inhibitor at 6 µM was needed to achieve 30% irreversible inhibition of *rh*AChE (Figure 3B). Thus, the irreversible inhibition of *Ag*AChE by 6 µM **AMTS13** is fast and nearly complete, while that of similarly treated *rh*AChE is slow and partial. The different time-courses offer additional evidence that the mechanisms for irreversible inhibition of the two enzymes are distinct.

### 2.4. Micelle Formation of AMTSn Detected by Atomic Force Microscopy

**Figure 4:**
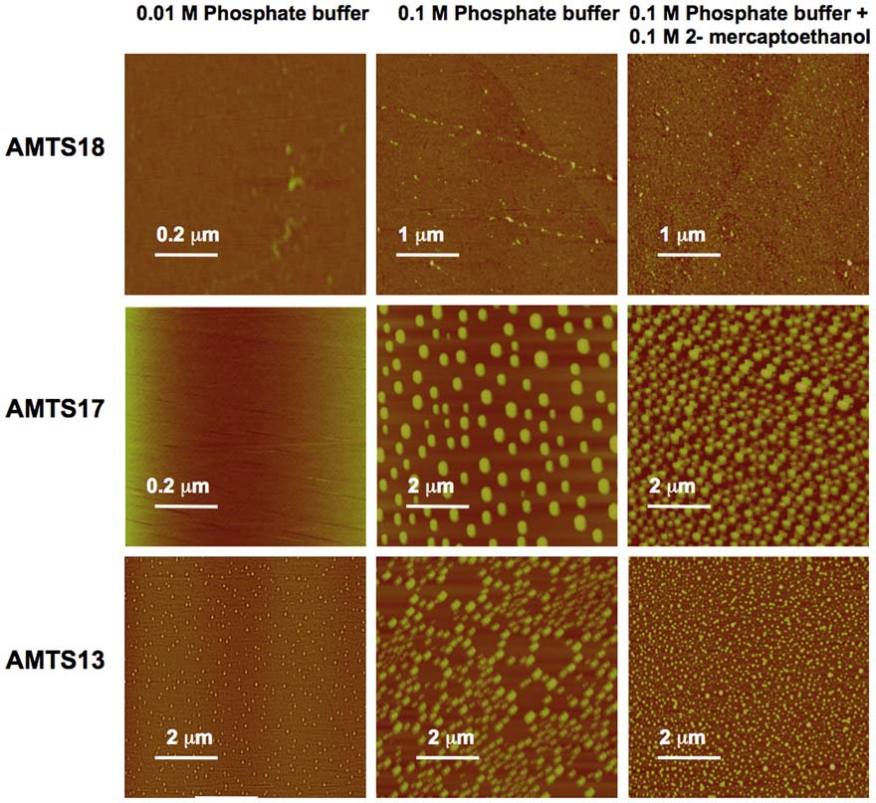
Atomic force microscopic images of AMTS18, AMTS17, and AMTS13 at different conditions.

One possible cause for the slow, partial, and irreversible inhibition of *h*AChE is partial enzyme denaturation induced by micelles of **AMTSn** that might form under the incubation conditions described in Section 2.1. We investigated this possibility with atomic force microscopy (AFM) [25], [26] and found that **AMTS13**, **AMTS17**, and **AMTS18** do not form micelles at 6 µM in distilled water. However, in samples with 100 mM sodium phosphate at pH 7.4, used in **AMTSn** incubation and for dialysis, AFM detected abundant micelles for **AMTS13** and **AMTS17** at 6 µM but no micelles for 6 µM **AMTS18** (Figure 4). Interestingly, **AMTS17** at 6 µM did not form micelles in 10 mM sodium phosphate at pH 7.4 (Figure 4). In contrast, **AMTS13** still formed detectable micelles at the same low ionic strength. A point relevant to the reactivation study described in Section 2.3 was that micelles of **AMTS13** did not disappear in the presence of 100 mM 2-mercaptoethanol, a condition that reversed the inhibitory effect of **AMTS13** on *Ag*AChE activity or on aphid AChE activity as reported previously [16]. These results further support that the fast, nearly full, and irreversible inhibition of *Ag*AChE is caused by the conjugation of **AMTS13** to the active-site Cys residue, and suggest that the slow, partial, and irreversible inhibition of *h*AChE is due to partial enzyme denaturation induced by micelles of **AMTS13**.

### 2.5. Circular Dichroism Spectroscopy Study on AMTS13-Treated rhAChE

**Figure 5:**
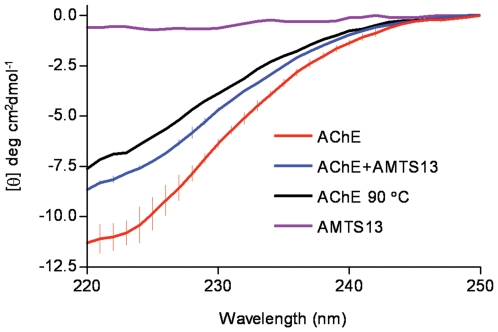
Circular dichroism spectra of AMTS13 and *rh*AChE at different conditions.

To test the inhibition mechanism for *h*AChE suggested by the AFM study, we acquired far-UV circular dichroism (CD) spectra for *rh*AChE at different conditions over the range of 220–250 nm in which a random coil or a nonpeptidic molecule results in zero absorption whereas an α helix and a β strand give rise to strong negative signals [27]. As shown in Figure 5, the *apo rh*AChE has the most negative absorption (−11.5 cm^−1^M^−1^) at 220 nm, consistent with reported CD spectra for AChEs from different species [28]–[30]. When the *apo rh*AChE was partially denatured by elevating temperature to 90°C, the absorption at 220 nm rose to −7.8 cm^−1^M^−1^ (Figure 5), indicating a reduction of ordered secondary-structural elements in the AChE structure. When 5 µM *rh*AChE was treated with a 100-fold molar excess of **AMTS13** for 48 hours, the absorption at 220 nm changed from −11.5 cm^−1^M^−1^ to −8.5 cm^−1^M^−1^ (Figure 5). The far-UV CD spectra clearly indicate that **AMTS13** reduces secondary structures or causes partial denaturation of *rh*AChE.

### 2.6. Crystal Structure of Mouse AChE in Complex with AMTS13

It is possible that the irreversible inhibition of *h*AChE could reflect not only partial denaturation, but also a reaction with the catalytic serine residue, as previously speculated [16]. To further investigate the latter possibility, we determined the X-ray crystal structure of a recombinant mouse AChE (*rm*AChE) in complex with **AMTS13** (**AMTS13**•*rm*AChE). Consistent with the AFM and CD results indicating partial denaturation of AChE, well-diffracting **AMTS13**-soaked crystals were difficult to obtain when using a protocol that has routinely yielded good quality crystals of *rm*AChE in complex with other reversible or irreversible inhibitors. The **AMTS13•** *rm*AChE crystals generally displayed high mosaicity and low resolution. After shortening the **AMTS13** exposure to two minutes, however, the mosaicity decreased, permitting acquisition of a dataset with a resolution of 2.6 Å (Table 2).

**Table 2:** Data Collection and Refinement Statistics.

Data collection	AMTS13•*rm*AChE
PDB entry code	2WLS
Wavelength (Å)	0.948
Space group	P2_1_2_1_2_1_
Unit cell dimensions (Å)	78.5×110.3×227.6
Resolution range (Å)	29.0−2.6 (2.74−2.6)
Total no. of reflections	450881 (65154)
Unique reflections	61580 (8866)
Completeness	99.8 (100.0)
Multiplicity	7.3 (7.3)
R_merge_ 1	0.083 (0.525)
Mean(I)/sd(I)	17.2 (4.4)
Refinement	
R-factor^2^/R_free_ 3	0.19/0.23
RMS bond lengths (Å)	0.008
RMS bond angle (°)	1.130
Ramachandran plot^4^	%/no. of residues
Favored regions	95.8/1015
Allowed regions	3.8/40
Outlier region	0.5/5

1 R_merge_ = (∑|I – <I>|)/∑I, where I is the observed intensity and <I> is the average intensity obtained after multiple observations of symmetry related reflections.

2 R-factor = (∑||F_o_|–|F_c_||)/∑|F_o_|, where F_o_ and F_c_ are observed and calculated structure factors, respectively.

3 R_free_ uses 2% randomly chosen reflections defined in Brunger [47].

4 The Ramachandran plots were determined using the program Rampage [52].

**Figure 6:**
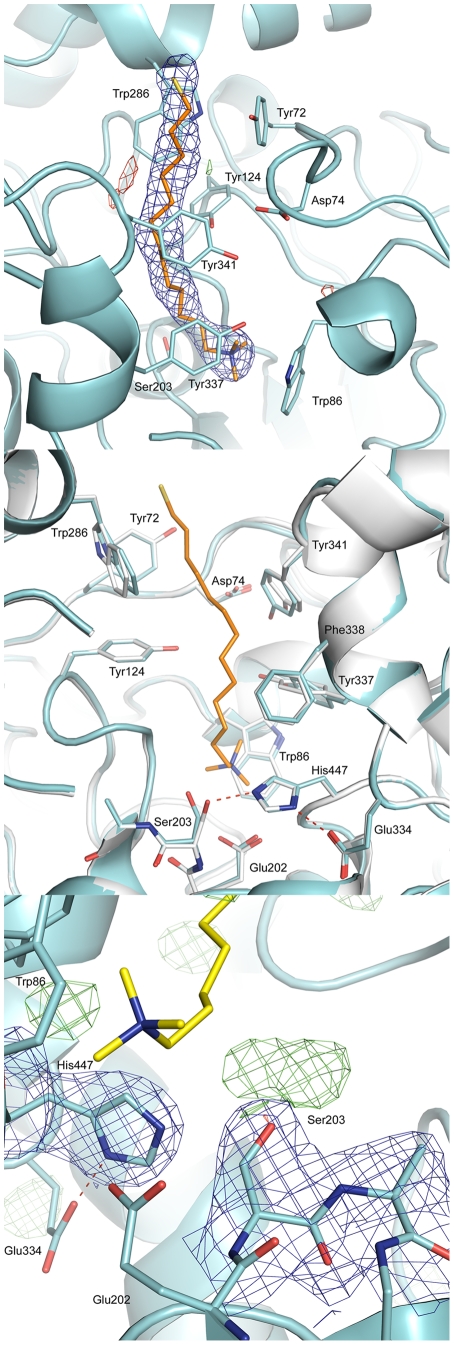
X-ray crystal structure of AMTS13•*rm*AChE (cyan) and the superimposed *apo rm*AChE crystal structure (grey). The 2|F_o_| – |F_c_| (1 σ) and |F_o_| – |F_c_| (3 σ) electron density maps are shown in blue and green, respectively.

The **AMTS13**•*rm*AChE structure resolved by this approach is generally similar to that of the *apo rm*AChE [31], with an alpha carbon root mean square deviation of 0.27 Å for monomer A. In the **AMTS13**•*rm*AChE crystal structure, the well-defined electron densities at the bottom of the active-site gorge reveal no sign of a covalent bond between **AMTS13** and Ser203. Instead, **AMTS13** spans the active-site gorge, with its linker adopting an extended conformation to interact with side chains of Tyr72, Tyr124, Trp286, Phe297, Phe338, and Tyr341, and with its ammonium group engaging in a cation-pi interaction with Trp86 (Figure 6A). The –SO_2_CH_3_ group was undefined as the electron density of the inhibitor is slightly disordered at the opening of the gorge (Figure 6). While this structure does not support the reaction with the catalytic serine residue, it does support the concept underlying our design strategy, namely that the ammonium group of **AMTS13** is near Trp86 and its methanethiosulfonate group is located in the acyl-loop region where the insect-specific Cys resides in mosquito AChEs.

## Discussion

### 3.1. Distinct Irreversible Inhibition Mechanisms for *Ag*AChE and *h*AChE

Before discussing results in relation to our primary goal, which was to determine whether or not the active-site Cys residue of *Ag*AP-AChE can be targeted selectively by sulfhydryl agents, it is necessary to consider mechanisms for the two distinct irreversible inhibitions of *Ag*AChE and *h*AChE by 6 µM **AMTS13** described in Sections 2.1–2.3.

### 3.1.1. Irreversible Inhibition through Disulfide Bond Formation

We deem it likely that the fast, nearly complete, and irreversible inhibition of *Ag*AChE by 6 µM **AMTS13** is initiated by a reversible interaction between the ammonium group of **AMTS13** and Trp84 of *Ag*AP-AChE, placing the methanethiosulfonate group at the rim of the gorge. This puts the reactive group close to the free Cys residue and facilitates a rapid conjugation of the inhibitor to *Ag*AP-AChE. Our **AMTS13**•*rm*AChE crystal structure supports the initial reversible inhibition. The subsequent conjugation step is evident from our observation that 2-mercaptoethanol caused significant reactivation of the **AMTS13**-inhibited *Ag*AChE. One may note that the 31% of **AMTS18**-inhibited *Ag*AChE activity restored by 2-mercaptoethanol was less than half of the previously reported 76% of **AMTS13**-inhibited aphid AChE activity [16]. This quantitative difference is consistent with the conjugation mechanism and can be explained by the sequence difference between aphid and mosquito AChEs (Table 3) as discussed below.

**Table 3:** The Sequence That Governs the Conformation of the Most Solvent-Exposed Disulfide Bond in Acetylcholinesterases of Different Species.

Species	Sequence
*Homo sapiens*	CPPGGTGGNDTELVAC
*Mus musculus*	CPPGGAGGNDTELIAC
*Schizaphis graminum*	CPDDRNTIHKTVEC
*Anopheles gambiae* sensu stricto	CPHEPSKLSDAVEC
*Torpedo californica*	CNLNSDEELIHC

**Figure 7:**
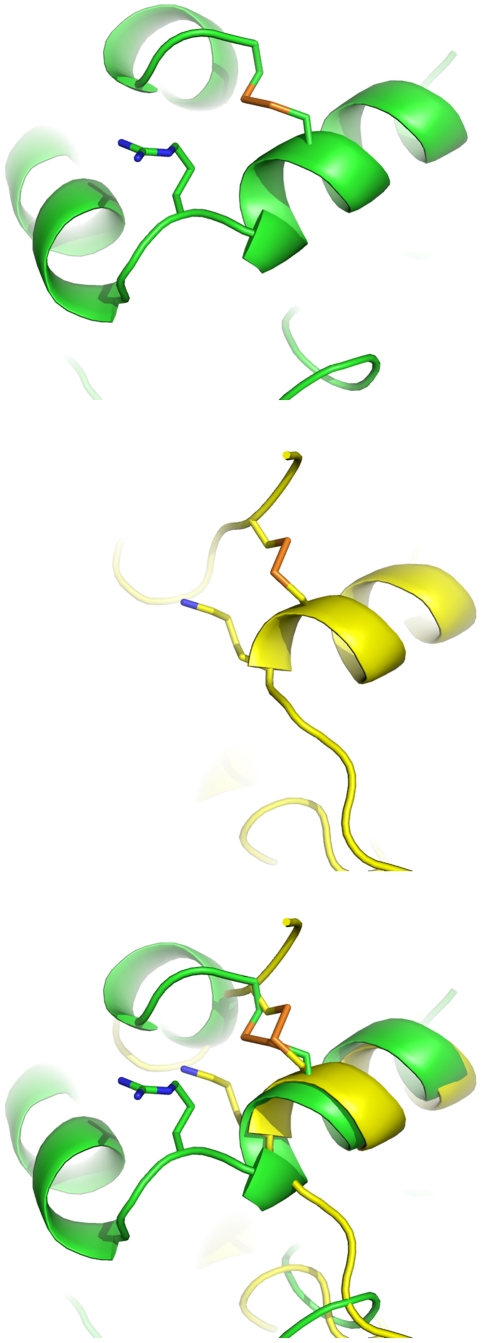
Structurally similar disulfide bonds in *rm*AChE (*green*) and α-lactalbumin (*yellow*).

In the *Torpedo californica* AChE crystal structure, Cys254-Cys265 is more exposed to solvent than Cys67-Cys94 and Cys402-Cys521 [19], [20]. It is known that Cys254-Cys265 can be ruptured readily by X-ray irradiation while the other disulfide bonds were either not affected or elongated by ∼0.7 Å under the same experimental condition [32], [33]. We found that Cys254-Cys265 or its equivalent in other AChEs is akin to Cys6-Cys120 of α-lactalbumin in terms of the location of the disulfide bond and the surrounding cationic residue (Figure 7), while Cys6-Cys120 is reportedly 140-times more reactive to reducing agents than normal disulfide bonds due to conformational strain at this locus [34]. We also found that the strain energy of the most solvent-exposed disulfide bond in mammalian AChE is about 0.6 kcal/mol higher than those of the others in mammalian AChE (Table 4), according to the density functional theory calculations using the coordinates of the **AMTS13**•*rm*AChE crystal structure and the procedure detailed in Section 4.10. It is conceivable that the most solvent-exposed disulfide bond in mammalian AChE is reactive to 2-mercaptoechanol and that variation in the sequence that governs the strain energy of the disulfide bond can change AChE's sensitivity to the reducing agent. Therefore, one should not expect 100% reactivation of the **AMTS13**- or **AMTS18**-inhibited insect AChE, nor, given the sequence variations shown in Table 3, should one expect the percentage of reactivation for *Ag*AChE to equal that for aphid AChE.

**Table 4:** Conformational Strain Energies of the Three Disulfide Bonds in the AMTS13•*rm*AChE Crystal Structure.

Disulfide Bond	Torsion* (° of arc)					Energy (kcal/mol)	
	X1	X2	X3	X2′	X1′	Conformational	Relative Strain
Cys257- Cys272	−62±13	−77±11	−62±5	−102±6	167±10	−1121502	11
Cys409- Cys529	−69±4	−46±0	−86±4	−37±1	−77±2	−1121508	5
Cys69- Cys96	−76±4	−45±2	86±2	130±7	63±4	−112513	0

* X1 = N-CA-CB-SG, X2 = CA-CB-SG-SG', X3 = CB-SG-SG'-CB', X2′ = SG-SG'-CB-CA, and X1′ = SG'-CB'-CA'-N'.

### 3.1.2. Irreversible Inhibition through Partial Enzyme Denaturation

Previously we speculated that the slow, partial, and irreversible inhibition of *h*AChE by 6 µM **AMTS13** could be caused by a reaction of **AMTS13** with the catalytic serine residue (Ser203), which is activated for nucleophilic reactions [16]. However, the **AMTS13**•*rm*AChE crystal structure at a resolution 2.6 Å reveals no sign of a covalent bond between **AMTS13** and Ser203, whereas a covalent bond between Ser203 and an organophosphonate or organophosphate is routinely observed under the same crystallographic conditions [35]–[38]. Moreover, the crystal structure shows that the methanethiosulfonate group is distant from Ser203. These findings are in agreement with our preliminary study using liquid chromatography triple quadrupole mass spectrometry. This study identified FGE**S**AGAASV (895.4 m/z), but FGE**S^S(CH2)13N+(Me)3^**AGAASV could not be identified, even in a sample of *rh*AChE that had been inhibited to a level of 70% by 72-hour treatment with **AMTS13** before pepsin digestion under acidic conditions. Therefore, the Ser203-involving mechanism now appears highly unlikely.

A more plausible inactivation mechanism in view of our studies using AFM and far-UV CD spectroscopy is partial denaturation of the enzyme induced by **AMTS13** and/or micelles of **AMTS13**. Two pieces of evidence for this mechanism are (1) the far-UV CD spectra that show a reduction of secondary structures of *rh*AChE treated with **AMTS13** for 45 hours and (2) the AFM results indicating **AMTS13**'s high propensity for micelle formation. The partial denaturation mechanism is further supported by the following considerations.

First, it has been reported that alkyltrimethylammonium halides with 12–16 methylenes, in particular dodecyl-trimethylammonium halides (DDTMAHs), are effective surfactants that can denature α-lactalbumin or β-lactoglobulin [39], [40]. Although methanethiosulfonate is slightly hydrophilic, the overall structure of **AMTS13** is analogous to DDTMAHs. While 6 µM **AMTS17** does not form micelles under low ionic strength condition and 6 µM **AMTS18** does not form micelles even under high ionic strength condition, 6 µM **AMTS13** forms micelles under both low and high ionic strength conditions. The relative propensities of **AMTS13**, **AMTS17**, and **AMTS18** for micelle formation agree with the reported range of the alkyl chain length for effective surfactants comprised of alkylene cations. In this sense **AMTS13** resembles DDTMAHs. Therefore, it is conceivable that **AMTS13** can fully or partially denature AChE just as DDTMAHs denature β-lactoglobulin [39].

**Figure 8:**
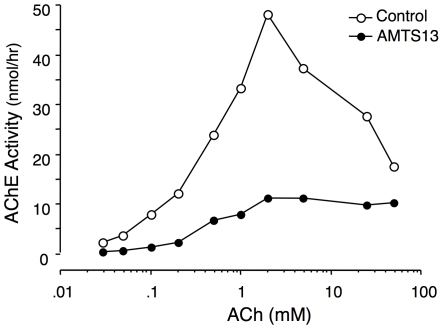
Substrate dependence of *h*AChE inhibition in extracts of human RBCs pretreated with AMTS13. Samples were exposed to 6 µM AMTS13 for 22 hours and dialyzed overnight before radiometric assay. Shown are residual *h*AChE activities as a function of substrate concentration in AMTS13-treated samples and paired controls.

Second, **AMTS13** has most of the structural features of DDTMAHs but, since it possesses a slightly hydrophilic methanethiosulfonate group, it cannot be quite so effective in denaturing proteins. The partial denaturing effect on AChEs that we ascribe to the **AMTS13** structure agrees very well with our observation that, after 22-hour exposure to **AMTS13**, inhibition of *h*AChE was inversely related to the concentration of acetylcholine (ACh) substrate in the enzyme assay, dropping from 85% at 1 µM ACh to 75% at 1 mM, 65% at 10 mM, and 57% at 50 mM. As apparent from Figure 8, substrate at high concentrations inhibited AChE in control enzyme preparations. This well-known effect [41] is presumably due to the binding of ACh at the peripheral “anionic” site. Strikingly, **AMTS13** exposure prevented the substrate inhibition. This outcome implies that **AMTS13** did not totally inactivate a subset of *h*AChE molecules but partially denatured most (or all) of them, depressing but not eliminating basic catalytic function. In addition, the loss of substrate inhibition suggests that **AMTS13** disturbed the peripheral “anionic” site.

Last, the posited partial denaturation also agrees with our time-resolved fluorescence decay study on native or heat-denatured *rh*AChE with or without treatment with **AMTS13** for 0, 24, and 48 hours. It is well-known that intrinsic Trp fluorescence in proteins is sensitive to local structural change induced by denaturation, ligand binding, or other means [42]. Since *rh*AChE has 13 Trp residues located in different local environments, we conducted studies on the intrinsic fluorescence lifetime (τ_i_) and its amplitude (α_i_). In our preliminary results (Table 5), the most noticeable change of fluorescence properties was that heating, treating with **AMTS13**, or extending the incubation time (with or without **AMTS13**) all led to an increase in α_1_. The largest increase (from 2.6% to 7.9%) occurred in the sample that was heated and treated with **AMTS13**. The second most noticeable change was that τ_1_, τ_2_, and τ_3_ of the heated enzyme were noticeably longer than those of the unheated ones. Finally, all changes of τ_1_ and α_1_ caused by **AMTS13** were, although lesser in extent, in the same direction as those caused by heating. In other words, the results are consistent with the mechanism that *rh*AChE underwent partial denaturation during treatment with **AMTS13**. Further studies could define how **AMTS13** denatures AChE. In our view, however, the present evidence, from multiple complementary approaches, is sufficient to establish a partial denaturation mechanism for the slow, incomplete, and irreversible inhibition of mammalian AChEs by **AMTS13**.

**Table 5:** Time-Resolved Fluorescence Intensity Decays of *rh*AChE at Different Conditions.

sample	τ_1_ (ns)	α_1_ (%)	τ_2_ (ns)	α_2_ (%)	τ_3_ (ns)	α_3_ (%)	χ^2^
*rh*AChE, 0 hr	0.280±0.110	2.6±0.3	2.091±0.065	43.6±1.1	5.015±0.054	53.9±1.4	0.85±0.03
*rh*AChE, 24 hr	0.135±0.025	2.8±0.3	1.985±0.042	42.2±1.0	4.839±0.036	54.1±1.2	0.79±0.06
*rh*AChE, 48 hr	0.422±0.033	3.8±0.1	2.251±0.015	49.3±0.7	5.235±0.037	47.0±0.8	0.90±0.01
*rh*AChE+ **AMTS13**, 0 hr	0.202±0.108	3.9±0.4	1.895±0.082	45.3±1.6	5.015±0.091	50.8±2.1	0.82±0.03
*rh*AChE+ **AMTS13**, 24 hr	0.189±0.092	3.3±0.1	1.847±0.067	47.0±1.5	4.610±0.090	49.7±1.6	0.78±0.01
*rh*AChE+ **AMTS13**, 48 hr^1^	0.390±0.037	4.4±1.5	1.999±0.093	49.6±2.1	4.769±0.129	46.9±3.7	0.85±0.01
*rh*AChE, 90°C^2^	0.494	5.8	2.529	44.0	6.346	50.2	1.02
*rh*AChE+**AMTS13**, 90°C^2^	0.480	7.9	2.439	45.3	6.172	46.8	0.97

1 Mean value±S.D. of two independent measurements at 20°C.

2 Single measurement of a 48 hours sample incubated at 90°C for 20 minutes prior to measurement.

Because **AMTS17** also forms micelles at 6 µM in buffers of high ionic strength, and its structure is at the borderline of surfactant classification, it is understandable why **AMTS17** does not cause significant irreversible inhibition of *h*AChE until its concentration reaches 100 µM. At the same time, it is worth emphasizing that partial denaturation does not apply to the fast, nearly complete, and irreversible inhibition of insect AChE by lower concentrations of **AMTS17** and its longer chain analogs. That is clear for three reasons. First, in 10 mM sodium phosphate, 6 µM **AMTS17** *did not* form micelles but irreversibly inhibited nearly 100% of *Ag*AChE (data not shown). Second, in 100 mM sodium phosphate, 6 µM **AMTS17** *did* form micelles but did not inhibit *h*AChE. Last, the irreversible inhibition of the *Ag*AChE was reversed by 100 mM 2-mercaptoethanol in 100 mM sodium phosphate, but these conditions did not prevent **AMTS17** from forming micelles.

### 3.1.3. Summary of the Two Distinct Irreversible Inhibition Mechanisms

We found the irreversible inhibition of *Ag*AChE by 6 µM **AMTS13** to be fast and nearly complete, and that of *h*AChE to be slow and partial. We explain the first inhibition as the conjugation of **AMTS13** to the active-site Cys residue of *Ag*AP-AChE, and the second as partial enzyme denaturation induced by **AMTS13**, free or in micelles. These mechanisms are attributed to two key features of the **AMTS13** structure: (1) a methanethiosulfonate group reactive toward free cysteine residues and (2) an alkyltrimethylammonium analogous to surfactant DDTMAHs.

### 3.2. Novel Mosquitocides for Controlling Mosquito-Borne Diseases

In the context of the irreversible inhibition mechanisms for insect and mammalian AChEs discussed above, the present work demonstrates that the mosquito-specific Cys residue at the entrance of the AP-AChE active site can be targeted by a strong sulfhydryl agent such as **AMTS17** or a weak alkylating agent such as **BPA11**. The present work also shows that the Cys residue can be targeted *selectively* by 1 µM **BPA11** or 6 µM **AMTS17**. Because 6 µM **AMTS17** has a low propensity for micelle formation and is thus unlikely to denature AO-AChE and mammalian AChEs, and because >80% AChE activity in mosquito is rapidly and irreversibly inhibited by **AMTS17**, we deduce that AP-AChE contributes most of the measurable AChE activity in the three studied mosquito species. It is true that mosquitoes possess both AP and AO AChEs and that irreversible AChE inhibition assays are more complicated than reversible inhibition assays. Nevertheless, all results presented in this article fit within a unified frame and support our view that the insect-specific Cys residue at the entrance of the AP-AChE active site in mosquito is a viable target.

We recognize that honeybees and silkworms also have AP-AChE with an analogous active-site Cys residue [14]. This fact raises a legitimate concern that Cys-targeting mosquitocides could harm these beneficial insects. Fortunately, there is an arginine residue residing on the rim of the AP-AChE active site conserved in several mosquito species but not found in other insects [14]. If AP-AChE inhibitors can be designed to target two mosquito-specific residues (*e.g*., Cys286 and Arg339 of *Ag*AP-AChE), it might be possible to reduce insecticide toxicity to mammals, fish, birds, and non-target insects that do not have the corresponding Cys and Arg residues. That would offer the prospect of controlling mosquito-borne diseases and alleviating resistance problems with reduced toxicity toward non-target species.

AChE is a well-studied enzyme and there is extensive literature on developing effective AChE inhibitors several of which are used currently as insecticides or even as clinical drugs for treating the Alzheimer's disease. It is therefore practical to develop the insect-specific insecticide concept illustrated in this article into an effective insecticide that targets the two mosquito-specific residues. Because insecticides are a well-established approach to controlling mosquito-borne diseases with a clear infrastructure and concept-of-use in the field, the insect-specific insecticides should enable rapid implementation of a cost-effective solution to controlling mosquito-borne diseases globally.

### 3.3. Conclusion

Under conditions that spare *h*AChE, low-concentration **AMTS17** is able to quickly, selectively, and irreversibly inhibit most of total AChE activity extracted from mosquitoes that transmit malaria, dengue, yellow fever, chikungunya, or St. Louis encephalitis. A much smaller but still selective and irreversible inhibition of mosquito AChE activity can also be achieved by a weak alkylating agent, **BPA11**. These inhibitors can slowly, partially, but also irreversibly inhibit human or mouse AChE at higher inhibitor concentrations. The mechanism for the fast, selective, nearly complete, and irreversible inhibition of mosquito AChE by long-chain compounds is their conjugation to the active-site Cys residue of mosquito AP-AChEs. The mechanism for the slow, nonspecific, partial, and irreversible inhibition of mammalian AChEs by short-chain and less selective agents is partial enzyme denaturation induced by the agents or by their detergent-like micelles. The present results imply that AP-AChE contributes most of the acetylcholine hydrolyzing activity in mosquitoes. They also demonstrate that the insect-specific free Cys residue at the entrance of the AP-AChE active site in mosquito can be targeted selectively by sulfhydryl agents with varied reactivity toward thiolate. Inhibitors targeting the insect-specific Cys residue and the mosquito-specific Arg residue hold promise in controlling mosquito-borne diseases and alleviating resistance problems with reduced toxicity toward non-target species.

## Materials and Methods

### 4.1. Mosquitoes and Chemicals

*An. gambiae* s. str. (G3, MRA-112) colonies obtained from the Malaria Research & Reference Reagent Resource Center (Manassas, VA) were maintained in the Department of Entomology at Kansas State University, and fourth-instar larvae were used in the study. Adult *Ae. aegypti* and *C. pipiens* were from the Department of Entomology of the University of Minnesota. Group AB human RBCs, bovine serum albumin (BSA), and *rh*AChE were obtained from Sigma-Aldrich (St Louis, MO, USA) as were 1,11-dibromoundecane, 4-(Boc-amino)pyridine, 2-mercaptoethanol, glutathione, acetylcholine iodide, pepsin, buffer constituents, and miscellaneous laboratory reagents. (±)-Dithiothreitol was purchased from Sigma-Aldrich (Buchs, Germany). Tritiated acetylcholine (99 mCi/mM) was purchased from New England Nuclear (Waltham, MA, USA). Cesium carbonate was obtained from AK Scientific (Mountain View, CA). Hexanes (Hex), ethyl acetate (EtOAc), and trifluoroacetic acid (TFA) were purchased from Fisher Scientific (Pittsburgh, PA) and used without purification. **AMTS7**–**AMTS20** were synthesized as previously described [16].

### 4.2. Synthesis of 11-bromo-*N*-(pyridin-4(1H)-ylidene)undecan-1-aminium trifluoroacetate (BPA11)

The ^1^H and ^13^C NMR spectra were recorded on a Varian Mercury 400 spectrometer. Chemical shifts are reported in ppm using either tetramethylsilane or the solvent peak as an internal standard. Data are reported as follows: chemical shift, multiplicity (s = singlet, d = doublet, t = triplet, q = quartet, m = multiplet), coupling constant, and integration. Medium pressure liquid chromatography (MPLC) was performed with Biotage SP-1 (Charlottesville, VA) using silica gel (EM Science, 230–400 mesh). Cesium carbonate (195 mg, 0.6 mmol) and 1,11-dibromoundecane (0.47 mL, 2.0 mmol) were added to a stirred solution of 4-(Boc-amino)pyridine (97 mg, 0.5 mmol) in anhydrous dimethylformamide (2 mL). After stirring for 7 hours at room temperature, the reaction mixture was diluted with water (2 mL) and extracted with EtOAc (2×50 mL). The combined organic extracts were washed with brine (3×5 mL), dried over MgSO_4_, and concentrated *in vacuo*. The residue was purified by MPLC (Hex:EtOAc/3∶2) to give *tert*-butyl 11-bromoundecyl(pyridin-4-yl)carbamate as oil (153 mg, 72%).^ 1^H NMR (400 MHz, CDCl_3_) δ 8.46 (m, 2H), 7.19 (m, 2H), 3.65 (t, *J* = 7.6 Hz, 2H), 3.37 (t, *J* = 6.8 Hz, 2H), 1.81 (m, 2H), 1.55 (m, 2H), 1.46 (s, 9H), 1.38 (m, 2H), and 1.23 (m, 12H). ^13^C NMR (100 MHz, CDCl_3_) δ 153.66, 150.44, 150.12, 119.14, 81.67, 48.98, 34.28, 33.00, 29.69, 29.60, 29.58, 29.40, 28.93, 28.69, 28.46, 28.35, and 26.91. This intermediate (153 mg, 0.36 mmol) was dissolved in 5 mL anhydrous dichloromethane followed by dropwise addition of 2 mL TFA and then stirred at room temperature for 3 hours. Removal of TFA and solvent *in vacuo* gave **BPA11** as a white solid (167 mg) in quantitative yield. **BPA11** used for *in vitro* testing was further purified by high performance liquid chromatography using a Phenomenex Gemini column (5 µm, C18, 250×21.20 mm), eluting with 50% of solution A (1000 mL of H_2_O and 1 mL of TFA) and 50% of solution B (100 mL of H_2_O, 900 mL of CH_3_CN, and 1 mL of TFA), and a flow rate of 10 mL/minute with a retention time of 24.60 minutes for **BPA11** (see Figures S1, S2, S3, S4 for proton NMR spectra and chromatograms of **BPA11** before and after the HPLC purification). ^1^H NMR (400 MHz, DMSO-*d*_6_) δ 13.01 (s, 1H), 8.51 (s, 1H), 8.18 (d, *J* = 6.4 Hz, 1H), 8.02 (d, *J* = 6.8 Hz, 1H), 6.82 (dd, *J* = 6.8, 14.4 Hz, 2H), 3.50 (t, *J* = 6.4 Hz, 2H), 3.22 (dd, *J* = 6.8, 12.8 Hz, 2H), 1.79–1.72 (m, 2H), 1.54–1.49 (m, 2H), and 1.32–1.23 (m, 14H); ^13^C NMR (100 MHz, DMSO-*d*_6_) δ 158.52, 141.44, 139.11, 110.43, 105.25, 42.77, 35.93, 32.90, 29.60, 29.54, 29.34, 28.77, 28.57, 28.18, and 26.96.

### 4.3. Measurement of AChE Inhibition

Mosquitoes were stored at −20°C, as were preparations of washed human RBC membranes prepared as described elsewhere [43]. For experiments involving exposure to test compounds and subsequent dialysis, one to five insects were homogenized twice for 10 seconds each in ground glass homogenizers containing 1 mL of an ice-cold solution of 0.09% NaCl, 0.1 M sodium phosphate at pH 7.4, 0.1% BSA, and Triton X-100 (0.5% v/v). RBC samples were prepared similarly but by sonication for 5 to 10 sec. In both cases the resulting fine suspensions were diluted 30-fold in the homogenization buffer before treatments and assays, and they were thoroughly re-suspended by vortex mixing as each aliquot was transferred to the reaction tubes. This procedure yielded duplicate agreements within±2% for mosquito AChE activity and within±1% for RBC AChE activity.

Experiments calling for sample exposure to inhibitors or reactivating reagents involved steps to separate the small molecules from the enzyme before determinations of AChE activity. Some data reported in this article were obtained after dialysis for 16–24 hours against two changes of 0.09% NaCl and 0.1 M sodium phosphate at pH 7.4 in more than 100-fold excess. This standard biochemical procedure was validated by control experiments demonstrating its ability to remove all traces of inhibitory activity as tested with sentinel samples of AChE. Time-course data were obtained by rapid inactivation of a test inhibitor using reduced glutathione (final concentration 500 µM).

The AChE assays were based on an established radiometric technique in which product (^3^H-labeled acetic acid) liberated enzymatically from substrate (^3^H-labeled acetylcholine, 50 nCi in a final reaction volume of 100 µL at pH 7.4) is partitioned into 4 mL of toluene-isoamyl alcohol (5∶1, v/v) with scintillation fluor [22], [44]. As a rule, the assays were performed with substrate in a concentration of 0.1 µM. This condition allowed maximum sensitivity (active samples more than 10 times the buffer-only blanks) with small samples (5 µg wet weight equivalent) and high temporal resolution (assay times as short as 5 min). Also, because of the low substrate concentrations a reversible 50% inhibition was expected to occur at concentrations near the true K_i_. When necessary, substrate concentration was adjusted by diluting stock material (99 mCi/mM) with unlabelled acetylcholine chloride. Assay duration, at room temperature, was rigorously controlled to ensure that signal was robust (>5 times blank value) and remained linear with respect to time and amount of sample present (typical conditions, 5 minutes for concentrated samples or up to 4 hours for highly dilute or low activity samples).

### 4.4. AChE Reactivation by 2-Mercaptoethanol

Reactivation by 2-mercaptoethanol was examined with AChE samples that were exposed for 1 hour to an inhibitor at 6 µM final concentration, followed by dialysis overnight against two changes of >100 volumes of 0.09% NaCl and 0.1 M sodium phosphate at pH 7.4. A few experiments (specifically indicated) used longer treatment times. Treated samples were exposed to 2-mercaptoethanol at a final concentration of 100 mM for 2 or 6 hr, followed by a second overnight dialysis against the phosphate buffer.

### 4.5. Atomic Force Microscopy

A Nanoscope IIIa SPM atomic force microscope (Digital Instruments, Inc., Santa Barbara, CA, USA) was used to detect micelle formation. About 0.5 mg of **AMTS13**, **AMTS17**, or **AMTS18** was dissolved in 1 mL of DMSO, and an aliquot (∼5.9 µL) of the stock solution was diluted to 6 µM with 1 mL of deionized water or 100 mM sodium phosphate (pH 7.4) with or without 100 mM 2-mercaptoethanol. To a freshly cleaved mica, 25 µL of **AMTS13**, **AMTS17**, or **AMTS18** at 6 µM was added, and the compound was allowed to bind onto the mica surface for three minutes. The unbound sample was removed by washing twice with deionized water (80 µL each), and the compound-bound mica was dried under a stream of argon. AFM images were collected using a tapping mode (http://www.veeco.com) with a high aspect ratio tip, Veeco Nanoprobe TM tips (Nanoscience Instruments Inc., Pheonix, AZ, USA). Each experiment was repeated three times. AFM images of the three repeated experiments for each compound showed similar sizes of micelles, and one representative image from the three experiments was shown in Figure 4.

### 4.6. Circular Dichroism Spectroscopy

*rh*AChE at 5 µM was treated with or without 500 µM **AMTS13** for 48 hours at 20°C in a buffer of 0.1 M sodium phosphate at pH 7.4. The circular dichroism spectra were obtained on a Jasco J810 spectrapolarimeter at 20°C using a path-length of 1 mm. Initial measurement in the 200–250-nm range revealed a rapid increase of the dynode voltage around 220 nm. However, a twofold dilution with water allowed measurement in the 220–250 nm range. Two measurements (each comprising eight scans) were performed for *rh*AChE treated with or without **AMTS13** at 20°C, whereas one measurement was made for the *apo rh*AChE heated to 90°C (70°C/hour).

### 4.7. X-Ray Crystallography

#### 4.7.1. Generation of *rm*AChE crystals

Cloning, expression, purification, and crystal screening of the *apo rm*AChE were performed as previously described [35]. An **AMTS13**-containing soaking solution was prepared by dissolving approximately 3 mg of **AMTS13** into 100 µL OX-buffer made of 28% (v/v) polyethylene glycol 750 monomethylether and 100 mM HEPES at pH 7.0. This was so that the concentration of **AMTS13** was much higher than the *K*_D_ of **AMTS13** for *rm*AChE. Crystals of *rm*AChE were first allowed to equilibrate slowly in OX-buffer at 20°C. The **AMTS13**-bound *rm*AChE complex was then generated by adding 3–5 µL of the **AMTS13**-containing soaking solution to the crystals. After incubation for ∼2 minutes the soaking was terminated by flash freezing the crystals in liquid nitrogen.

#### 4.7.2. Collection, processing, and refinement of diffraction data

The X-ray diffraction data were collected at the MAXlab synchrotron centre (Lund, Sweden) using beam line I911-3 on a MAR Research CCD detector. The images were collected with an exposure time of 60 sec, an oscillation angle of 1.0° per exposure, and the total oscillation range covered 180°. Intensity data were indexed and integrated with XDS [45] and scaled using Scala [45]. The **AMTS13**-bound *rm*AChE structure was determined using rigid-body refinement on a modified structure of *apo rm*AChE (Protein Data Bank entry code: 1J06 [31]) as a starting model. To avoid model bias, Ser203, Trp286, and all small-molecule ligands were removed from the starting model. Further crystallographic refinement was carried out using the program Refmac5 and phenix.refine [46]. The refinement included 98% of the data while the remaining 2% was used to follow the progress of the refinement with R-free [47]. The R-free dataset was obtained from a previously determined structure (Protein Data Bank entry code: 2GYU [35]). In the initial electron density map of the **AMTS13**-bound *rm*AChE complex structure, a clearly visible electron density feature spans the active-site gorge of *rm*AChE. Due to the intermediate resolution of 2.6 Å (Table 2), several rounds of refinement were performed with consideration of two orientations of **AMTS13** relative to the *rm*AChE active site. One orientation is with the **AMTS13** ammonium group located close to Trp86 at the bottom of the gorge, while the other is with the ammonium group positioned close to Trp286 at the rim of the gorge. During the progress of the refinement, it became apparent that a cation-pi interaction between the ammonium group and the indole ring of Trp86 is consistent with the crystallographic data. During the refinement, a model was rebuilt manually after visualization of 2|F_o_| – |F_c_| and |F_o_| – |F_c_| maps using the COOT program [48]. Water molecules were added using COOT, phenix.refine, and by manual model building. The angles and bonds for the sulfhydryl group were determined using the Gaussian98 program [49] and the Hartree-Fock method with the 6-31G* basis set. The quality of the final model was evaluated using PROCHECK, WHATCHECK, and Rampage [50]–[52]. Figures were made using the PyMol program [53].

### 4.8. Time-Resolved Fluorescence Spectroscopy

In all fluorescence measurements the excitation wavelength was set to 295 nm to record only tryptophan emission and to exclude interference of phenylalanine or tyrosine as an emitting species. The *rh*AChE concentration was kept at 1.7 µM, a level that corresponded to an O.D. of 0.04 at 295 nm, which was low enough to avoid self-absorption processes. Background fluorescence from buffer solutions with 500 µM **AMTS13** was always checked. All measurements were performed at room temperature. The time-resolved measurements with resolution down to sub-nanosecond time regime were performed with time-correlated single-photon counting (TCSPC) technique by using an IBH spectrometer equipped with a data station hub, TBX-04 photon detection module, light emitting diode Nanoled-17 (HORIBA Jobin Yvon IBH) generating 295 nm UV-light pulses of 1 MHz repetition rate, and a 5000 M single-grating emission monochromator. The fluorescence emission was collected at 340 nm where the monochromator spectral bandwidth was adjusted to 16 nm. An instrumental response function was obtained using suspension of silica particles (Ludox, Aldrich) in deionized water, which typically gave a spectral width of about 600 ps at half maximum. The fluorescence were measured over 4096 channels (13 ps per channel), and fluorescence lifetimes (τ_i_) and pre-exponential factors, presented as amplitudes (α_i_), were calculated by fitting experimental decays either with double or triple exponential functions using the IBH DAS6 decay analysis software (such functions are based upon least-squares fitting algorithms and reconvolution with the experimental response function). The tri-exponential fits typically gave χ^2^ in the range of 0.8–0.9, low enough to be acceptable (those fits obtained with double exponential functions typically displayed χ^2^ around 0.9–1.0). Typically, the fluorescence decay was measured and the mean±S.D. was calculated for three independent samples.

### 4.9. Liquid Chromatography Triple Quadrupole Mass Spectrometry

Glutathione was reduced using (±)-dithiothreitol and subsequently reacted with **AMTS13** to obtain **AMTS13**-glutathione. With the aid of a triple quadrupole mass spectrometer (Waters/Micromass Quattro, Milford, MA, USA), the conjugated glutathione was verified and abundant fragments were determined through collision-induced dissociation. One abundant fragment has 272.3 *m/z* that corresponds to 13-(trimethylammonio)tridecane-1-thiolate. After treating *rh*AChE with **AMTS13** for 72 hours, the enzymatic activity was reduced by 72%. This partially inhibited enzyme was then split into two portions. One was digested using pepsin under acidic conditions and the resulting peptides were separated and analyzed using liquid chromatography mass spectrometry (PepMap100, C18, 3 µm, 100 Å, 150×0.075 mm, LC Packings, Netherlands; capLC pump, Waters/Micromass, Milford, MA, USA). For the other, free **AMTS13** was removed (Spin-filter Microcon Ultracel YM-10, Millipore, Bedford, MA, USA) prior to the pepsin digestion.

### 4.10. Density Functional Theory Calculations

The Gaussian98 program [49] was used to compute the strain energies of the three disulfide bonds in the **AMTS13**•*rm*AChE crystal structure. The coordinates of two cross-linked cysteine residues were taken from the **AMTS13**•*rm*AChE crystal structure (Protein Data Bank entry code: 2WLS); the amino and carboxylate groups were masked by –COMe and –NHMe, respectively; the entire system was fully optimized in vacuum using the B3LYP method with the 6-311+G(d,p) basis set; frequency calculations were performed after the energy minimizations to confirm the minimized structures were at their minima (*viz*, no imaginary frequency was found in the frequency calculation).

## Supporting Information

Figure S1 Proton NMR spectrum of BPA11 before HPLC purification
(0.25 MB PDF)

Figure S2 Proton NMR spectrum of BPA11 after HPLC purification
(0.25 MB PDF)

Figure S3 Analytical chromatogram of BPA11 before HPLC purification
(0.18 MB PDF)

Figure S4 Analytical chromatogram of BPA11 after HPLC purification
(0.20 MB PDF)
