# Supplementary figures and images for: Selective and Irreversible Inhibitors of Mosquito Acetylcholinesterases for Controlling Malaria and Other Mosquito-Borne Diseases
Source: PLoS One. 2009 Aug 28;4(8):e6851. doi: 10.1371/journal.pone.0006851 (PMC2731169; doi:10.1371/journal.pone.0006851)

# BPA11 before HPLC

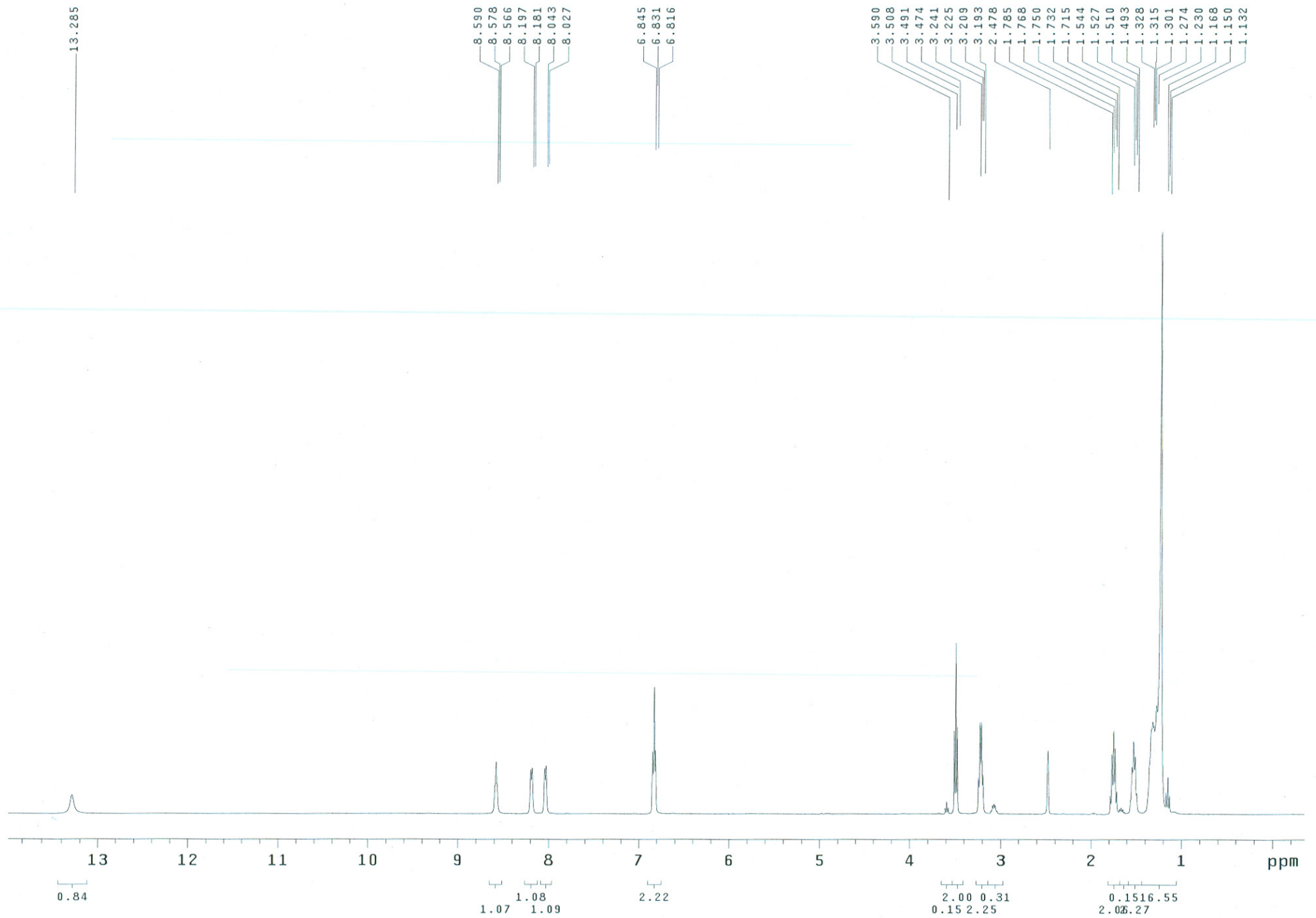

Supplement: Figure S1 — Proton NMR spectrum of BPA11 before HPLC purification (0.25 MB PDF) [file pone.0006851.s001.pdf]

# BPA11 after HPLC

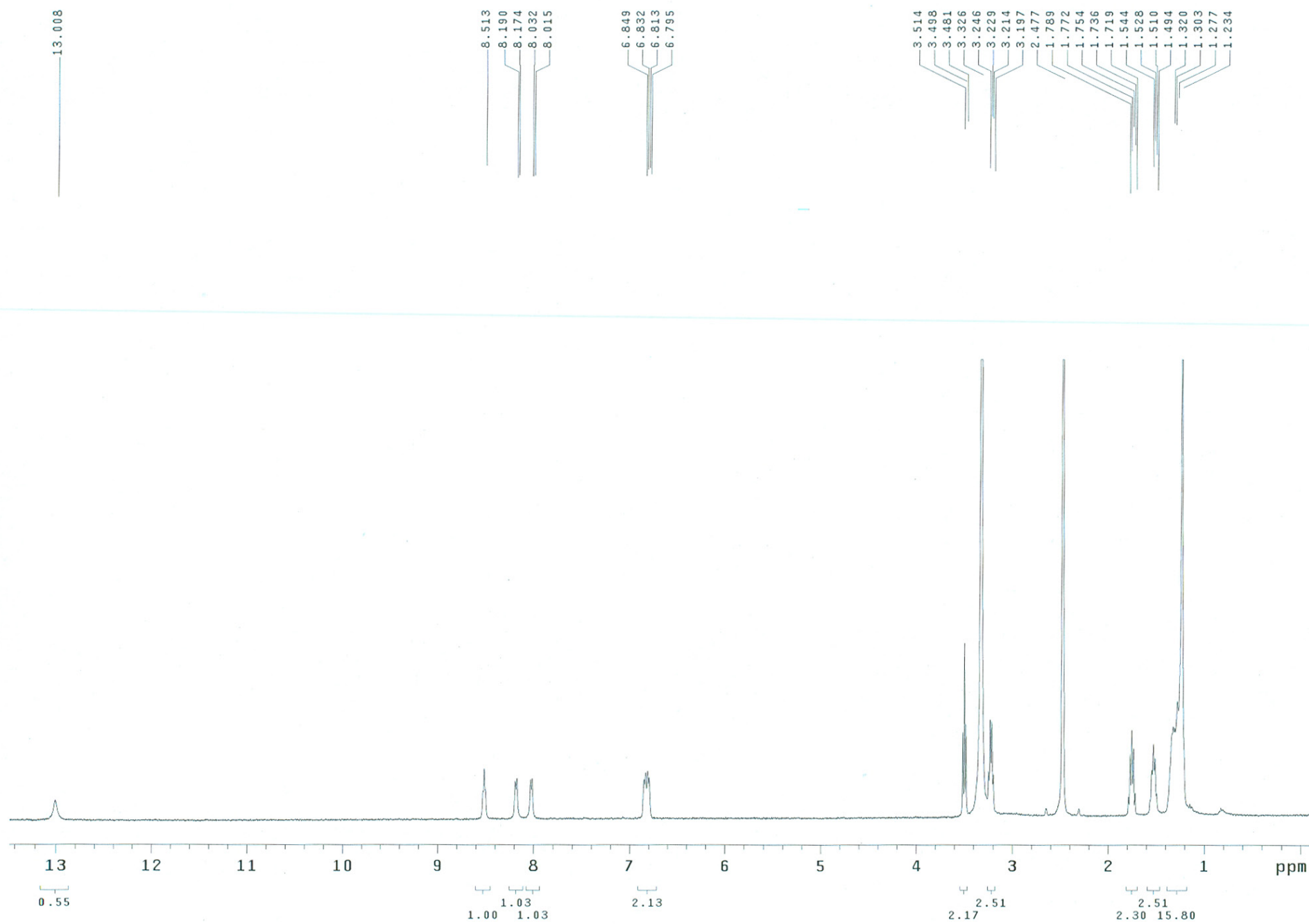

Supplement: Figure S2 — Proton NMR spectrum of BPA11 after HPLC purification (0.25 MB PDF) [file pone.0006851.s002.pdf]

**BPA11 before HPLC**

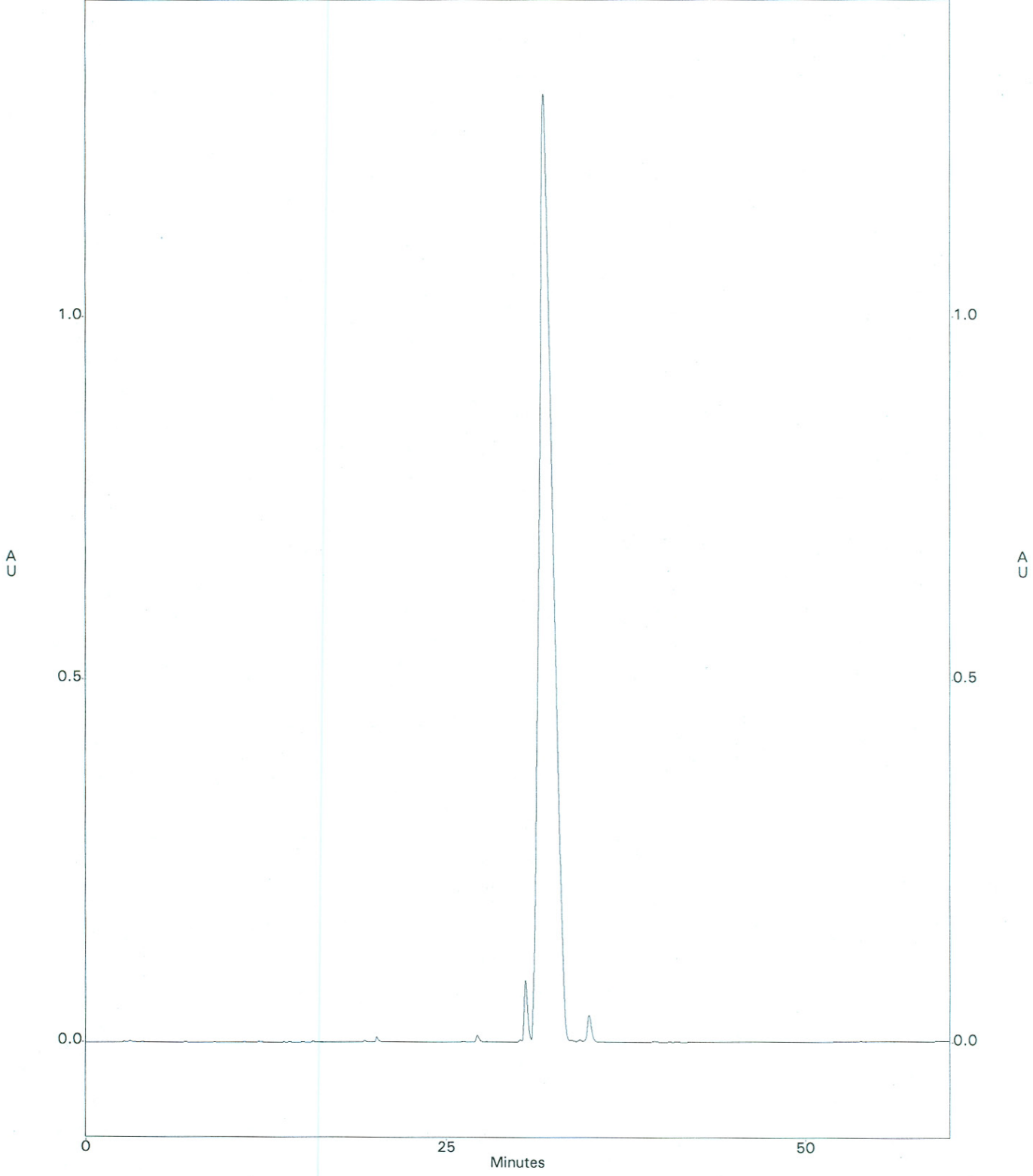

Supplement: Figure S3 — Analytical chromatogram of BPA11 before HPLC purification (0.18 MB PDF) [file pone.0006851.s003.pdf]

# BPA11 after HPLC

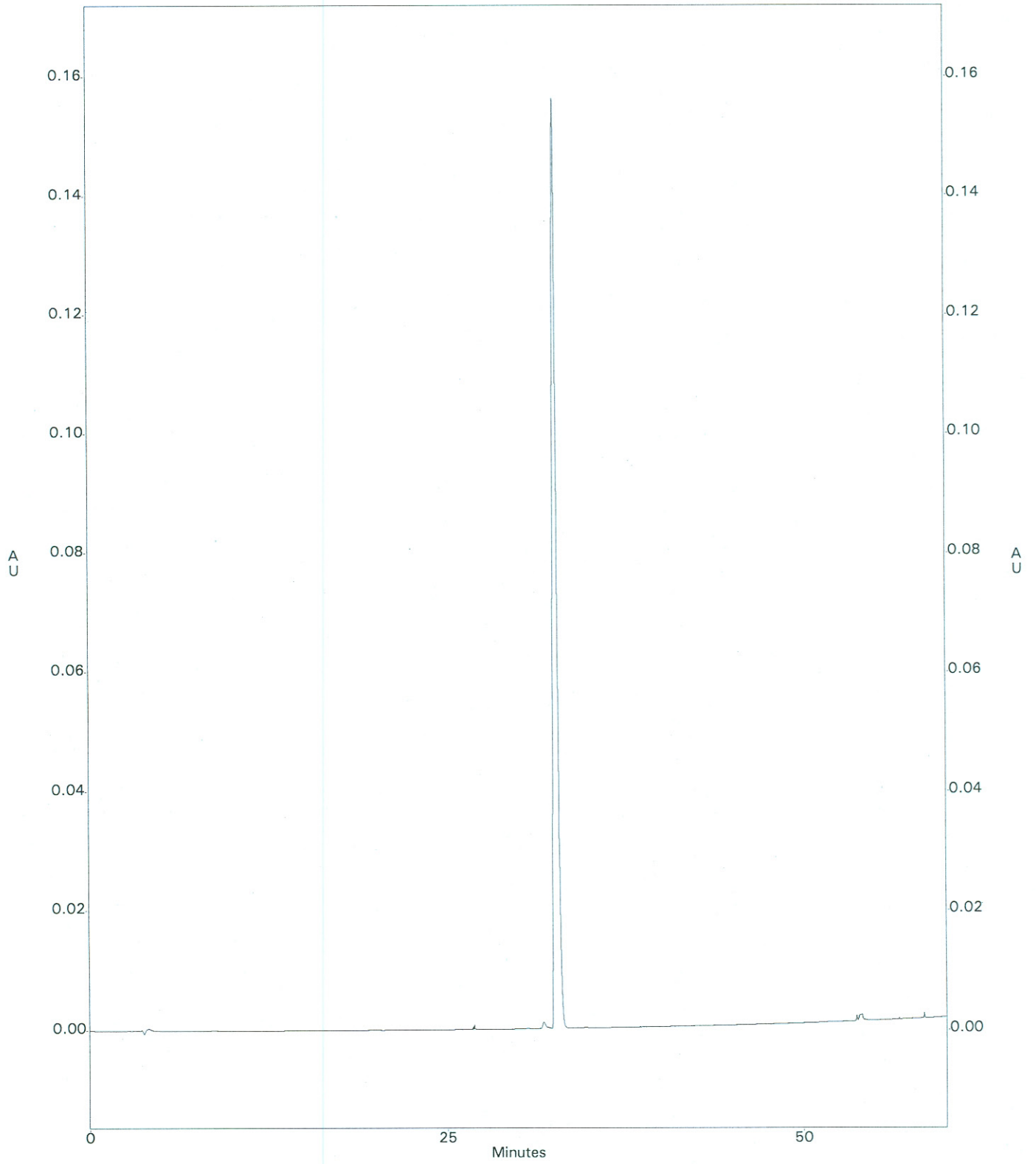

Supplement: Figure S4 — Analytical chromatogram of BPA11 after HPLC purification (0.20 MB PDF) [file pone.0006851.s004.pdf]
